# Trace Detection of Di-Isopropyl Methyl Phosphonate DIMP, a By-Product, Precursor, and Simulant of Sarin, Using Either Ion Mobility Spectrometry or GC-MS

**DOI:** 10.3390/toxics13020102

**Published:** 2025-01-28

**Authors:** Victor Bocoș-Bințințan, Paul-Flaviu Bocoș-Bințințan, Tomáš Rozsypal, Mihail Simion Beldean-Galea

**Affiliations:** 1 Faculty of Environmental Science and Engineering, Babeș-Bolyai University, RO-400294 Cluj-Napoca, Romania; 2 Transcend SRL, RO-400568 Cluj-Napoca, Romania; paulbocos@gmail.com; 3 Nuclear, Biological and Chemical Defence Institute, University of Defence, Vita Nejedleho 1, 682 03 Vyskov, Czech Republic; tomas.rozsypal@unob.cz; 4 Raluca Ripan Institute for Research in Chemistry, Babeş-Bolyai University, RO-400294 Cluj-Napoca, Romania

**Keywords:** di-isopropyl methyl phosphonate (DIMP), ion mobility spectrometry (IMS), trace detection, simulants for chemical warfare agents (CWAs), sarin (GB), soman (GD), gas chromatography with mass spectrometry (GC-MS)

## Abstract

Di-isopropyl methyl phosphonate (DIMP) has no major commercial uses but is a by-product or a precursor in the synthesis of the nerve agent sarin (GB). Also, DIMP is utilized as a simulant compound for the chemical warfare agents sarin and soman in order to test and calibrate sensitive IMS instrumentation that warns against the deadly chemical weapons. DIMP was measured from 2 ppb_v_ (15 μg m^−3^) to 500 ppb_v_ in the air using a pocket-held ToF ion mobility spectrometer, model LCD-3.2E, with a non-radioactive ionization source and ammonia doping in positive ion mode. Excellent sensitivity (LoD of 0.24 ppb_v_ and LoQ of 0.80 ppb_v_) was noticed; the linear response was up to 10 ppb_v_, while saturation occurred at >500 ppb_v_. DIMP identification by IMS relies on the formation of two distinct peaks: the monomer M·NH_4_^+^, with a reduced ion mobility K_0_ = 1.41 cm^2^ V^−1^ s^−1^, and the dimer M_2_·NH_4_^+^, with K_0_ = 1.04 cm^2^ V^−1^ s^−1^ (where M is the DIMP molecule); positive reactant ions (Pos RIP) have K_0_ = 2.31 cm^2^ V^−1^ s^−1^. Quantification of DIMP at trace levels was also achieved by GC-MS over the concentration range of 1.5 to 150 μg mL^−1^; using a capillary column (30 m × 0.25 mm × 0.25 μm) with a TG-5 SilMS stationary phase and temperature programming from 60 to 110 °C, DIMP retention time (RT) was ca. 8.5 min. The lowest amount of DIMP measured by GC-MS was 1.5 ng, with an LoD of 0.21 μg mL^−1^ and an LoQ of 0.62 μg mL^−1^ DIMP. Our results demonstrate that these methods provide robust tools for both on-site and off-site detection and quantification of DIMP at trace levels, a finding which has significant implications for forensic investigations of chemical agent use and for environmental monitoring of contamination by organophosphorus compounds.

## 1. Introduction

The investigated compound DIMP (di-isopropyl methyl phosphonate) is an organophosphorus (OP) chemical. DIMP is a liquid with no major commercial uses and is a by-product when the nerve chemical warfare agent sarin (code GB) is synthesized; it constitutes ca. 20% of raw sarin. Moreover, the nerve agent sarin could be manufactured starting from DIMP, using a one-step high-yield methylphosphonoyldichloride preparation using SOCl_2_ chlorination [1]; therefore, DIMP is a precursor of sarin.

Since it has a chemical structure and physico-chemical properties very similar to those of nerve chemical warfare agents, but also a far lower acute toxicity, DIMP is utilized very often as a simulant (surrogate) compound for G-type nerve chemical warfare agents sarin (GB) and soman (GD), in order to calibrate the fast, sensitive IMS instrumentation that detects and warns against these deadly nerve chemical weapons. Being involved in sarin production, either as a by-product or as a raw material (precursor), one may reasonably assume that detection of DIMP traces indicates, in an indirect manner, that the nerve agent sarin (GB) is present as well. Table 1 summarizes the main characteristics of DIMP, while Table 2 compares the most relevant properties of DIMP vs. the nerve agents sarin (GB) and soman (GD).

From Table 2, it is easy to observe that DIMP has, actually, the closest similarity with the nerve agent soman (GD).

DIMP may enter the organism as vapors, either through the skin or through the respiratory system (lungs), having, therefore, certain toxicity for humans and mammals; its toxic action is shown by a series of symptoms specific to acute intoxications by OP chemicals. However, the acute toxicity of DIMP is medium/low, since oral lethal doses are between 500 and 1500 mg kg^−1^, depending on the species (Table 2).

The primary goal of this paper is the fast detection of DIMP vapors in the air, at ultra-trace levels (well below 1 ppm_v_), using ToF (time-of-flight) ion mobility spectrometry (IMS) as a fast, ultra-sensitive instrumental technique based on the soft ionization of chemicals at atmospheric pressure. IMS has witnessed a continuous, spectacular development over the last decades and has a range of advantages, such as an outstanding sensitivity, a remarkably short response time (seconds), a large number of applications, and the use of miniaturized instruments that are operated at atmospheric pressure. The gold standard GC-MS has also been employed, mainly as a confirmation analytical technique.

Detection and quantification of organophosphorus compounds—either extremely toxic nerve CWAs and their simulants (surrogate compounds) or various OP pesticides—which present as vapors in air samples are currently conducted by using a variety of analytical methods, such as colorimetric techniques, photoionization detectors (PID), gas chromatography coupled with mass spectrometry (GC-MS), ion mobility spectrometry (IMS), SAW (surface acoustic wave) sensors, or flame photometry detectors (FPD) [3]. Nevertheless, the most employed field technology is currently ion mobility spectrometry [4].

Ion mobility spectrometry (IMS) is well known, nowadays, as being a fast and highly performant analytical technology, widely applied for the trace identification of vapors of a large number of compounds present in air samples—but also in liquid or solid samples—after their soft ionization at atmospheric pressure [4]. Given the fact that it measures ionic currents in the picoamperes range, IMS possesses an excellent sensitivity, with detection limits of low ppb_v_ for many chemicals. The rapidity of ion separation at atmospheric pressure in the gas phase is, probably, to be viewed as the main advantage of IMS, since a single spectrum is usually acquired in just twenty milliseconds, a quality which couples brilliantly with the already mentioned outstanding sensitivity in the low-ppb_v_ range for many classes of chemicals and without any pre-concentration. Moreover, the simple operation of IMS instrumentation, which has dramatically evolved after its inception in the 1970s toward ultra-compact miniaturized and rugged devices, has resulted in the well-deserved popularity of IMS instruments—like the rapid sensing of explosives [5,6], illegal drugs and their precursors [7,8], or toxic industrial chemicals [9,10,11,12], toward microorganism sensing and discrimination [13,14,15] or toxicological applications [16]. IMS technology has also been applied in many other fields, like forensic analysis, bio-medicine, industrial hygiene, space investigation, and protection of the environment [4].

IMS has an immense versatility, because it is capable of quickly detecting and quantifying a very large range of compounds, both inorganic and organic. As a general principle, any chemical that can be ionized can then be sensed using IMS. We must emphasize too that IMS is clearly a green analytical tool: because it does not use any reagents or external gases, IMS does not generate any waste.

Ion mobility spectrometry has, certainly, many similarities with several instrumental and separation techniques. The most striking similarities are those between IMS and mass spectrometry (especially ToF-MS and CI-MS) and with chromatographic techniques as well. In the positive ionization mode, IMS and photoionization detection (PID) present similarities concerning ionization mechanisms and the speed of response; the main difference is that PID lacks the separation of positive cations.

IMS is based upon two successive steps: (a) ionization of neutral analytes, a process which takes place in the gaseous phase and at atmospheric pressure, followed by (b) the separation of ions previously generated, based on their different ionic mobilities—which depend on both ion mass and size—in a low-intensity d.c. electric field (E < 500 V/cm). This initial variant of IMS, in which the ions are separated using a longitudinal electric field and the response (the ion mobility spectrum) is just a representation of the analytical signal (ion current) against the travelling (drift) time, is currently known as the time-of-flight (ToF) IMS or drift tube (DT) IMS. After the advent of ToF IMS in the 1970s, other variants of ion-mobility-based techniques have emerged—such as aspiration-type IMS (a-IMS) and differential mobility spectrometry (DMS). In ToF IMS, the separation of the ions takes place because they possess different mobilities through a neutral drift gas—usually recirculated purified air—under the moving force manifested by the longitudinal d.c. electric field applied onto the IMS cell. When an ionization source is used in the air, a complicated sequence of fast ion–molecule processes produces, first, a series of ions, known as reactant (or primary) ions. In the positive ion mode and by using water vapor chemistry, reactant ions are cluster ions of type (H_2_O)_x_H^+^ (predominant), (H_2_O)_y_NH_4_^+^, and (H_2_O)_z_NO^+^, while the predominant negative reactant ions are the (H_2_O)_n_O_2_^−^ species. After the reactant ions are formed, they transfer the electrical charge to molecules of target analyte(s) and, this way, the product ions are generated. Because water vapor plays a paramount role in the whole ion–molecule chemistry at atmospheric pressure, its concentration must be carefully controlled inside the IMS cell and kept at a constant and low level [9,10]. In order to obtain the optimum analytical performance, it is best to operate the ion mobility spectrometer using, as drift gas, relatively dry air that contains only several ppm of water vapor; in that case, the water clustering degree of ions is limited and, most importantly, kept constant over time.

In the positive ion operation mode, the IMS technology is most suitable for those compounds possessing high proton affinity (such as nerve chemical warfare agents), while, in the negative ion operation mode, those chemicals with high electron affinity (for instance, explosives or blister CWAs) are sensed.

## 2. Materials and Methods

### 2.1. The IMS Instrument

A pocket-sized commercial portable ToF IMS instrument (ca. 18.0 × 11.5 × 4.5 cm and with a weight of ca. 0.6 kg), model LCD-3.2E (Smiths Detection Ltd., Watford, UK), was used for detection and quantification of DIMP vapors. Its miniature IMS cell uses a classic design with stacked rings (alternate discrete conducting elements being placed on the insulating tube of the cell), with a drift length of ca. 30 mm and an electric field intensity E of ca. 270 V cm^−1^. With two physical drift cells operated in parallel (“twin cell” design), this IMS system generates, simultaneously, both positive and negative ion mobility spectra. The temperature of operation was ca. 25 °C and the measured pressure inside the IMS cell was ca. 1000 mbar. The ionization source was a non-radioactive one, using a point-to-place corona discharge. A corona discharge is a viable alternative to the radioactive sources using beta isotopes (like 63-Ni or 3-H) that are employed in the vast majority of ToF IMS instruments in operation today. With an ionization chemistry very similar to that of radioactive sources, a source based on corona discharge produces a higher signal (usually by an order of magnitude). The drift gas was dry air, circulated through a closed-loop pneumatic circuit including the filter based on a molecular sieve. Note that the same filter continuously delivers low levels of gaseous ammonia (NH_3_), which is a dopant that enhances selectivity in the positive ion mode. This IMS instrument is amongst the smallest commercial ToF IMS systems that currently exist and is able to sense both TICs and CWAs very quickly, within seconds; it has been described in detail (including a schematic diagram) in reference [12].

The IMS instrument was operated via a computer, through the proprietary software TrimScan2, ver. 0.4.0 (Smiths Detection Ltd., UK). The experimental data (as positive and negative IMS spectra) were saved on the hard disc of the PC and then they were examined using the same TrimScan2 software before being exported to MS Excel files.

As a principle, IMS relies on the separation of ions, in the gas phase and at atmospheric pressure, based on the differences in the drift speed of ions in a constant, low d.c. electric field. Because the molecules of the target analyte are not fragmented, the ionization process is a soft one and it occurs in two stages: the formation of reactant ions, then the generation of product ions (which contain the entire molecule of the analyte) by fast charge transfer from reactant ions to the analyte molecule. The formation of the so-called “reactant ions” is the first stage; these ions are the clusters (H_2_O)_n_H^+^, which prevail when water vapor chemistry is used, plus lower amounts of (H_2_O)_m_NH_4_^+^ and (H_2_O)_u_NO^+^ (in the positive operation mode) or (H_2_O)_n_O_2_^−^ (in the negative operation mode). When ammonia is used as a dopant, clusters of (H_2_O)_m_NH_4_^+^ become the major positive reactant ions. When a small air sample with DIMP is sent into the IMS cell, the non-radioactive ionization source, using the corona discharge, ionizes the molecules of DIMP in a soft manner, so that they remain unfragmented and, eventually, form product ions that include the whole molecule of the target analyte. All ions—both reactant and product—then travel through the “drift length” (which extends from the shutter grid to the detector) and reach a constant drift speed of several m s^−1^. When the ions—both reactant ions and product ions—strike the detector, they produce each an ion current (picoamperes) that is amplified and measured. Every substance has a distinct drift speed in the neutral drift gas; as a consequence, the drift time can be used for identification, in a similar manner as the retention time in chromatography.

Ion mobility, K, represents the constant that connects the drift speed v_d_ of an ion to the electric field intensity E that moves that ion along the drift length of the IMS cell: v_d_ = K·E = l_d_/t_d_, where l_d_ is the drift length of the IMS cell and t_d_ is the drift time of a certain ion. Further, ion mobility may be expressed as K = v_d_/E = l_d_/(E·t_d_). Reduced ion mobility, K_0_, which is widely used as a qualitative parameter for a certain compound [4], is actually the ion mobility K normalized for both operating temperature and pressure inside the measurement cell: K_0_ = K·(T_ambient_/T_cell_)·(P_cell_/P_atmospheric_).

### 2.2. The GC-MS System

A Thermo Scientific GC-MS, model Thermo Focus DSQ II (Thermo Fisher Scientific, Austin, TX, USA), which includes the GC Thermo Focus coupled with a Thermo Quadrupole DSQ II Dual Stage Quadrupole MS, was used.

A narrow-bore capillary column (Thermo Fisher Scientific) with a 30 m length × 0.25 mm i.d. × 0.25 μm thickness of stationary phase film (low polarity, TraceGoldTM TG 5 SilMS) was used. The carrier gas (ultra-pure He) flowrate was 1.2 mL min^−1^, at a constant flow; the GC inlet and the MS transfer line were kept at 200 °C; the temperature programming consisted of a start at 60 °C, kept for 1 min, then a raise to 110 °C during the following 10 min (ramp increase: 5 °C min^−1^). The parameter of the MS detector was a scan mode—full scan, positive ions, mass range—from 50 to 250 Da. The run time of the GC was set to 12 min.

Methodology: a total of 1 μL of standard solution with 150 μg mL^−1^ DIMP in methanol was injected into the GC-MS, then various split ratios were used to change the on-column mass—from 1:1 (mass injected: 150 ng) to 100:1 (mass injected: 1.5 ng).

### 2.3. Reagents, Sampling, and Work Flow Procedure

Liquid DIMP with a purity of 90% (Thermo Electron, USA) was used without purification.

For all GC-MS experiments, a known volume of liquid DIMP was dissolved into HPLC grade methanol, and then this stock solution was diluted to a standard solution containing 150 μg mL^−1^.

For all IMS measurements, standard atmospheres with known low concentrations of DIMP vapors were generated by a dynamic method that used a series of vapor sources based on the permeation of DIMP through a PTFE polymeric membrane with a thickness of 0.1 mm and an area of ca. 12 mm^2^, thermostated at a constant temperature of 50 °C and calibrated gravimetrically using a laboratory ultra-microbalance with a resolution of 10^−5^ g, model Sartorius ME-235S.

Using the permeation vapor sources, seven different standard (test) atmospheres containing known low concentrations of DIMP vapors were prepared and further investigated using the ToF IMS instrument model LCD-3.2E: 2 ppb_v_, 5 ppb_v_, 10 ppb_v_, 30 ppb_v_, 100 ppb_v_, 200 ppb_v_, and 500 ppb_v_. Table 3 summarizes the experimental conditions used for generating the abovementioned standard atmospheres.

## 3. Results

### 3.1. IMS Analysis

The experimental data produced by the IMS instrument model LCD-3.2E as ion mobility spectra were sequentially recorded for each of the seven concentrations of DIMP. The measurements for each concentration level were realized in triplicates and standard deviations between 6% and 2% were observed.

The results of our investigation are summarized in Table 4, where C_DIMP_ is the concentration of DIMP in the standard atmosphere (see Table 3).

The ion mobility spectrometric response consisted of characteristic spectra, with two product ion peaks (PIP) noticed in the positive ion mode, at drift times of t_d_ = ca. 7.6 ms (for the monomer DIMP·NH_4_^+^) and t_d_ = ca. 10.3 ms (for the dimer DIMP_2_·NH_4_^+^). The positive reactant ion peak (POS RIP) was observed at a drift time of t_d_ = ca. 4.7 ms.

**Figure 1:**
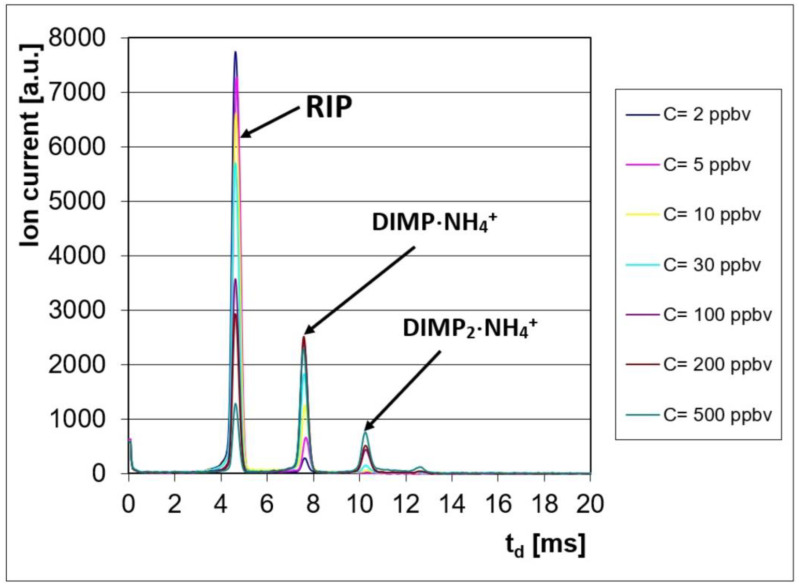
Ion mobility spectra from DIMP, obtained in the positive ion mode.

These ion mobility spectra obtained in the positive ion mode are presented in Figure 1; they clearly show the conservation of electrical charge: when the DIMP vapor concentration increased, the intensity of the positive RIP (reactant ion peak) decreased, while, simultaneously, the intensity of the product ion peaks produced by DIMP increased.

We must also mention that DIMP did not produce an IMS response in the negative ion mode.

**Figure 2:**
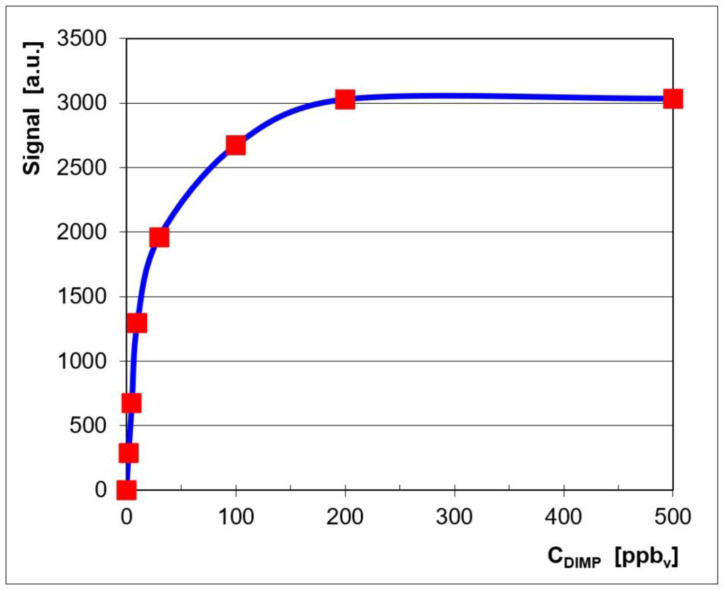
Calibration for DIMP, in the positive ion mode.

Quantitative data were also plotted in order to get the associated calibration curve and to assess the quantitative response of the LCD-3.2E IMS to vapors of DIMP. This calibration curve is displayed in Figure 2, where the signal (ion current) is the sum of the currents of both the monomer product ion and the dimer product ion.

The IMS spectra contained both qualitative information that was based on the specific drift time (t_d_) of an ion (and, hence, on the reduced mobility (K_0_) of that ion) and quantitative information that resided in the peak height. The drift time was relatively proportional to the mass and size of the ions and inversely proportional to the ion’s electrical charge. Summarizing and simplifying, any ion mobility peak in the spectrum can be described by using three numbers: (1) its drift time, t_d_ (given in milliseconds, ms), (2) its reduced mobility, K_0_ (in cm^2^ V^−1^ s^−1^), and (3) its peak amplitude (height), h_max_ (in a.u.).

Qualitative information, which included the reduced ion mobilities for the positive reactant ion peak and for the DIMP product ion peaks (monomer and dimer), is summarized in Table 5. It is worth mentioning here that DIMP generated a pair of product ion peaks, a monomer and a dimer, which is a feature characteristic of all OP compounds.

Reduced ion mobility (K_0_) was calculated using the so-called “IMS cell constant”; this method has the immense advantage of taking into account even the smallest non-homogeneities in the d.c. electric field (E) that carries ions through the IMS cell. The use of this cell constant also excludes the necessity of accurately measuring both instrumental parameters (drift length, l_d_, and electric field intensity, E) and environmental parameters (temperature and pressure in the IMS cell). In the negative ion operation mode, methyl salicylate (MSAL) is widely used as a chemical standard for ion mobility, having a known reduced ion mobility of K_0 of standard (MSAL)_ = 1.474 cm^2^ V^−1^ s^−1^ [9], while, in the positive mode, 2,4-lutidine (2,4-dimethylpyridine) is widely accepted as a chemical standard, with reduced ion mobilities of K_0 of standard (Lutidine monomer)_ = 1.95 cm^2^ V^−1^ s^−1^ and K_0 of standard (Lutidine dimer)_ = 1.43 cm^2^ V^−1^ s^−1^, respectively [17]. The ion mobility cell constant (noted with A) represents, in fact, the product between the reduced mobility (K_0_) and the drift time (t_d_) of the chemical standard ion. In other words, in order to calculate the reduced mobility of DIMP peaks, the simple relationship A = K_0 of standard (Lutidine dimer)_·t_d of standard (Lutidine dimer)_ = K_0 of analyte (DIMP)_·t_d of analyte (DIMP)_ was used. Since t_d of standard (Lutidine dimer)_ = 7.47 ms, then the cell constant was A = 1.43 cm^2^ V^−1^ s^−1^·7.47·10^−3^ s = 10.682·10^−3^ cm^2^ V^−1^.

The ratio between the drift times of PIPs and RIP, equivalent to the ratio of their afferent reduced mobilities, was t_d PIP 1_/t_d RIP_ = K_0 RIP_/K_0 PIP1_ = 1.636 for the monomer product ion DIMP·NH_4_^+^ and t_d PIP 2_/t_d RIP_ = K_0 RIP_/K_0 PIP2_ = 2.215 for the dimer product ion DIMP_2_·NH_4_^+^. In fact, this ratio represents a normalization of the product ions’ drift time against the drift time of the positive reactant ion peak.

The resolving power of the LCD-3.2E IMS instrument, R_IMS_, is defined as the ratio between the drift time of a certain ion and its width at half height (R_IMS_ = t_d_/Δt_d_) and was calculated for all the peaks present in the ion mobility spectrum; the results are shown in Table 6.

These values obtained for the resolving power—between 15 and 30—are usual for commercial hand-held IMS instruments equipped with miniaturized measure cells, like the LCD-3.2E instrument.

#### Validation

A very simple and rapid validation process was performed in order to evaluate the suitability of the developed analytical method. The assessed parameters were the limit of detection, limit of quantitation, sensitivity, range of linear response, accuracy, and trueness of the proposed method using ion mobility spectrometry.

The limit of detection (LoD) is defined as the lowest concentration that produces a signal-to-noise (S/N) ratio equal to 3, while the limit of quantitation (LoQ) is defined as the lowest concentration that generate an S/N ratio of 10. Sensitivity (S) is defined as the change in signal Y (namely, peak height) that follows when the concentration is modified (S = ΔY/ΔC). The background signal—described as the standard deviation (s_d_) of the background noise—was obtained using the last 500 data points (signals obtained at drift times from 10.00 to 20.00 milliseconds, in increments of 0.02 milliseconds) for all IMS spectra corresponding to blanks and to each investigated concentration of the analyte; this average value was found to be s_d_ = 10.3 a.u. in the positive ion mode. Table 7 presents the figures of merit concerning DIMP detection; the IMS response over the linear range (≤10 ppb_v_ DIMP) was considered.

Precision was assessed by using analyses in triplicates (see Table 3). Accuracy was evaluated by using the relative standard deviation (RSD, called also coefficient of variation CV), which was between 2% and 6% for the PIP in the positive ion mode. Good repeatability of results was noticed, with an RSD < 10%.

### 3.2. GC-MS Analysis

**Figure 3:**
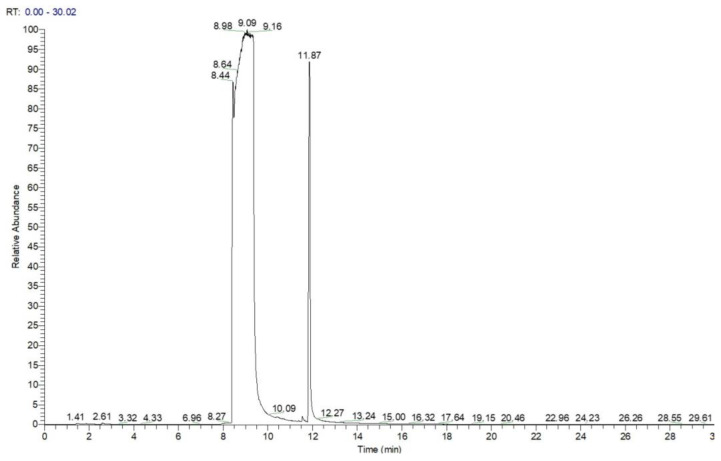
Chromatogram for DIMP—1 μL of 60 mg mL^−1^, splitless. Note the peak of the impurity tri-isopropyl phosphate (TIPP) at a retention time of ca. 12 min.

Analysis by GC-MS for the highly concentrated standard solution with DIMP, containing 60 mg mL^−1^ in methanol (1 μL injected, splitless), produced a chromatogram that clearly shows the massive overloading with the analyte (Figure 3). The large peak with a retention time of ca. 9 min was found to correspond, using the confirmatory mass spectrum, to the target analyte DIMP, while the peak with a retention time of ca. 12 min was identified by MS as being tri-isopropyl phosphate (TIPP), having the formula C_9_H_21_O_4_P and a molecular weight of M = 224 Da; CAS #513-02-0. This compound is most probably the main impurity existent in the liquid standard of DIMP, which is stated by the manufacturer to possess a purity of just 90% DIMP.

Chromatograms were further obtained using a diluted standard solution, containing just 150 μg mL^−1^ of DIMP in methanol. A volume of 1 μL from this solution was injected in the GC-MS instrument, and several successive split ratios were used in order to decrease the on-column mass: for 1:1 (splitless), the mass of DIMP injected was 150 ng; for a 10:1 split, the DIMP mass was 13.64 ng; for a 50:1 split, the DIMP mass was 2.94 ng; for a 100:1 split, the DIMP mass was 1.49 ng.

**Figure 4:**
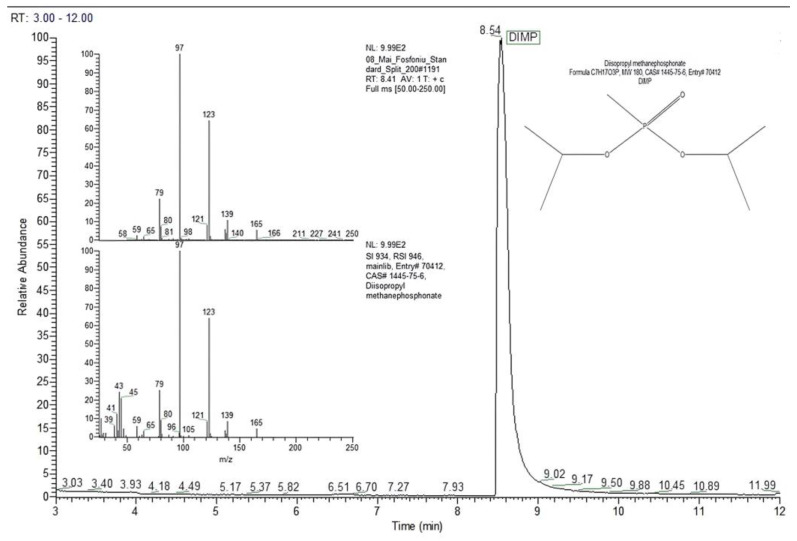
Chromatogram and mass spectrum for DIMP, for an on-column mass of 13.64 ng (split ratio 10:1).

Figure 4 presents the chromatogram generated by 13.64 ng of DIMP (split ratio 10:1), together with the mass spectrum for the DIMP peak with a retention time of 8.41 min.

The mass spectrum for DIMP includes a set of characteristic peaks that appeared (in order of ions abundance) at the following mass-to-charge ratios: *m*/*z* 97 (base peak, 100%); *m*/*z* 123 (ca. 65%); *m*/*z* 79 (ca. 24%); *m*/*z* 139 (ca. 10%); *m*/*z* 165 (ca. 5%).

The chromatographic peak with a retention time of 8.54 min was assigned by the MS NIST library to DIMP (C_7_H_17_O_3_P, MW 180 Da, CAS# 1445-75-6) with a probability of almost 97%.

#### Validation

The limit of detection (LOD) and the limit of quantification (LOQ) of the GC-MS instrument were determined by statistical approaches, using the standard deviation (SD) of the regression line and the slope (S) of the calibration curve using the following equations: LOD = 3.3 σ/S and LOQ = 10 σ/S [18]. The figures of merit for the GC-MS system are summarized in Table 8.

The calibration curve was built using standards of 15.00, 7.50, 3.75, 1.88, and 0.94 µg mL^−1^, obtained by successive dilution of a stock standard solution with 150 µg mL^−1^ DIMP in methanol. Precision was assessed using analyses in triplicates.

## 4. Discussion

The logarithmic-type appearance of the calibration curve (Figure 2) is, in fact, characteristic of an IMS response produced using a classical radioactive ionization source [4]. One may, therefore, notice the close resemblance of the quantitative IMS response from the pocket-held ToF IMS instrument, equipped with a non-radioactive ionization source, which was used by us (the LCD-3.2E) and the response produced by other ToF IMS devices that have a radioactive source.

The IMS spectra for higher concentrations of DIMP (>200 ppb_v_) in the positive ion mode show that the saturation threshold was almost reached, since, at 500 ppb_v_, the reactant ion peak was still present at only ca. 15% of its initial value. Saturation means that the whole batch of reactant ions was consumed; the consequence was the disappearance of the reactant ion peak from the IMS spectrum. It has to be emphasized that the saturation of the ion mobility spectrometer must be avoided, because it will usually lead to a persistent contamination of both IMS measurement cell and all inner surfaces that came in contact with the analyte; this contamination could finally produce totally unwanted memory effects.

The possible interferences from other chemicals are always a concern, and this is, of course, an issue for IMS instruments as well. However, because DIMP produces, at concentrations levels of just tens of ppb_v_, two peaks in the positive ion mode (the monomer and the dimer), the qualitative identification of DIMP using two time windows simultaneously is, therefore, much more reliable compared to that using a single peak.

Examination of the quantitative response (Figure 2) and of the ion mobility spectra obtained for all the investigated concentration levels (Figure 1) allows us to say that:

1. The minimum measured concentration of DIMP vapors was just 2 ppb_v_ (0.015 mg m^−3^). For OP compounds, this level produces mild physiological effects such as miosis (constriction of the eye pupil).
2. The linear dynamic range was from 2 ppb_v_ (0.015 mg m^−3^) to ca. 30 ppb_v_ (0.225 mg m^−3^) DIMP.
3. Saturation as estimated to appear at >500 ppb_v_ (3.75 mg m^−3^) DIMP; this finding is in good agreement with the fact that the dynamic range of an ion mobility spectrometer extends by about two orders of magnitude.
4. The IMS peak that appeared at the highest DIMP concentration with t_d_ = 12.64 ms and K_0_ = 0.845 cm^2^ V^−1^ s^−1^ may be most probably assigned to the impurity of tri-isopropyl phosphate, which is present in the liquid DIMP with 90% purity used by us and was identified by GC-MS.

All the IMS responses for DIMP were obtained in the positive ion mode, because DIMP does possess a high proton affinity (PA). Vapors of DIMP did not generate any spectrum in the negative ion mode. The pocket-held instrument model LCD-3.2E responded in real time (in just several seconds) to vapors of DIMP.

The net identity of both reactant and product ions produced in the IMS cell can be assigned, with a decent degree of conclusiveness, by coupling the IMS cell with a mass spectrometer, which is able to identify the formed ions. These hyphenated IMS-MS systems have been employed already, with the aim of finding the real identity of ions generated especially by explosives, illicit drugs, chemical warfare agents, or highly toxic chemicals like chlorine and phosgene [9,10]. Assigning the identity of ions produced by DIMP vapors inside the IMS cell (LCD-3.2E) was, therefore, not feasible in this study. However, it was previously found that, in IMS systems using NH_3_ as a dopant and at temperatures < 50 °C (as it is the case with the LCD-3.2E instrument), the monomer and dimer product ions are ammoniated species, not protonated ones; the assignation of product ions from the DMMP compound (another surrogate of nerve agents) was made using mass spectrometry [19]. As a consequence, we assume that both product ions formed by DIMP inside the IMS cell were ammoniated species.

Reduced ion mobilities for the monomer and dimer product ion peaks generated in the positive mode were found to be K_0_ = 1.41 cm^2^ V^−1^ s^−1^ for the monomer DIMP·NH_4_^+^ and K_0_ = 1.04 cm^2^ V^−1^ s^−1^ for the dimer DIMP_2_·NH_4_^+^; both values are in good compliance with other results found in the literature (see Table 9). Literature information concerning DIMP detection and quantification using various IMS instruments and that found by us is summarized in Table 9, where LoD is the limit of detection and LoQ the limit of quantification. One may observe that most references provide only qualitative information regarding DIMP sensing (reduced ion mobilities, K_0_, for monomer and dimer product ions); with this study, we intended to offer a quantitative insight as well.

ToF IMS instruments with water vapor ionization chemistry produced protonated product ions (protonated monomer DIMP·H^+^ and proton-bound dimer DIMP_2_·H^+^) with reduced ion mobilities (K_0_) of ca. 1.53 cm^2^ V^−1^ s^−1^ (for monomer ion) and ca. 1.09 cm^2^ V^−1^ s^−1^ (for dimer ion) (Table 8). Using a ToF IMS instrument with a different ionization chemistry (doped with ammonia) means that the product ions are not protonated species anymore, but ammoniated ones that have the form of DIMP·NH_4_^+^ and DIMP_2_·NH_4_^+^. If this is the case, the mass of an ammoniated product ion is larger (by approx. 9%) than the mass of a similar protonated product ion; consequently, the reduced ion mobility of ammoniated monomer ions will be smaller than that of protonated monomer ions by ca. 9%. Therefore, for the monomer, we have K_0 ammoniated monomer_ = 0.906 × K_0 protonated monomer_ = 0.906 × 1.55 = 1.40 cm^2^ V^−1^ s^−1^ (while our finding was 1.41 cm^2^ V^−1^ s^−1^), while for the dimer we have K_0 ammoniated dimer_ = 0.953 × K_0 protonated dimer_ = 0.953 × 1.09 = 1.04 cm^2^ V^−1^ s^−1^ (while our finding was 1.04 cm^2^ V^−1^ s^−1^).

Ammonia doping of the IMS cell has the crucial advantage of preventing competitive ionization from a very large number of chemicals in the positive ion mode; therefore, selectivity is greatly enhanced, since any compound having a proton affinity smaller than that of ammonia (854 kJ mol^−1^) will not be able to “steal” the positive charge from the positive reactant ions. On the contrary, DIMP (with a PA of 931 kJ mol^−1^) forms easily positive product ions as ammoniated clusters.

The GC-MS measurements performed within this study are in very good agreement with the literature. Similar retention times of 7–10 min for GC columns of 30 m × 0.25 mm × 0.25 μm and nonpolar stationary phases such as Zebron 5MS, with a temperature program (initial temperature 40 °C (1 min), rate 10 °C/min, final temperature 280 °C for 5 min), were observed [28]. The mass spectrum of the chromatographic peak of DIMP included the base peak at *m*/*z* 97, which indicates the presence of the fragment ion [CH_6_PO_3_]^+^ (formed when DIMP loses both i-propyl groups). This base peak at *m*/*z* 97 and the peak at *m*/*z* 79 (formed by losing a water molecule from an ion with *m*/*z* 97) are characteristic of dialkyl methylphosphonate compounds. The peak at *m*/*z* 123 most probably formed when DIMP lost both the i-propyl and the methyl group.

Although we have a known minimum measured amount of DIMP by GC-MS (1.5 ng DIMP), since the volume of air sampled by the pocket-held ToF IMS model LCD-3.2E is not known, it is quite difficult to compare IMS with GC-MS. However, if we assume that the volume of air sampled for a single IMS measurement is just 1 mL, and since the lowest measured DIMP vapor concentration sensed by IMS was 0.015 mg m^−3^, then the minimum mass of DIMP that elicited a response in IMS was only 0.015 ng. Comparing these two minimum masses, we could estimate that the IMS instrument LCD-3.2E is about 100 times more sensitive than the GC-MS system. 

## 5. Conclusions

Fast detection and quantification of di-isopropyl methyl phosphonate (DIMP), which is simultaneously a G-nerve CWA surrogate compound and a by-product and precursor of the chemical warfare agent sarin, was performed at ultra-trace levels (from low ppb_v_ to hundreds of ppb_v_) using a portable, lightweight, pocket-held ToF IMS instrument with non-radioactive (corona discharge) ionization, source model LCD-3.2E (Smiths Detection Ltd.). DIMP vapors were successfully detected and quantified in real time (seconds) in the positive ion mode. Characteristic spectra that included two product ion peaks, both assignable to DIMP, with reduced ion mobilities of K_0_ = 1.41 cm^2^ V^−1^ s^−1^ (assignable to monomer product ion DIMP·NH_4_^+^) and K_0_ = 1.04 cm^2^ V^−1^ s^−1^ (assignable to dimer product ion DIMP_2_·NH_4_^+^), were obtained. DIMP identification by IMS can, therefore, be achieved using these two product ion peaks present in the ion mobility spectrum.

For the IMS system, a seven-point calibration curve for DIMP vapors—from 2 to 500 ppb_v_ (0.015 to 3.75 mg m^−3^)—was built. The detection limit was found to be LoD 0.24 ppb_v_ (1.8 μg m^−3^) DIMP, while the limit of quantification was LoQ 0.80 ppb_v_ (6.0 μg m^−3^) DIMP. Saturation of the IMS response is expected to appear at concentration levels above 500 ppb_v_ DIMP. The minimum measured concentration of DIMP was 2 ppb_v_, which is the concentration level where OP compounds produce the mildest physiological effects, namely miosis.

We successfully demonstrate through this study that low-resolution, pocket-held ToF IMS instrumentation with a non-radioactive (corona discharge) ionization source is admirably fit for rapid sensing and quantification of vapors of DIMP at ultra-trace levels, while providing a series of other strategical advantages—such as sensitivity, sensibility, and real-time responses—compared with other analytical techniques.

The second goal of this work concerns the detection and quantification of DIMP using the gold standard—the tandem GC-MS (gas chromatography coupled with mass spectrometry). The chromatograms for a series of on-column masses were obtained, between 1.5 ng and 150 ng of DIMP. Using a narrow-bore capillary column (30 m long, 0.25 mm i.d., 0.25 μm stationary phase width) with a low-polarity stationary phase type (TG-5 SilMS) and a temperature program from 60 to 110 °C, DIMP analysis by GC-MS was performed in a relatively short time—less than 10 min. The identification of DIMP was successfully realized using the mass spectrum of DIMP. The limit of detection was LoD 0.21 μg mL^−1^, and the limit of quantification was LoQ 0.62 μg mL^−1^ DIMP. Moreover, the main impurity present in the DIMP standard (90% pure) was found to be tri-isopropyl phosphate.

Detection of DIMP as a simulant compound of both sarin (GB) and soman (GD) in the field, in real time, and at ultra-trace levels (low parts per billion, ppb_v_) is highly recommended to be accomplished by using IMS as the analytical technique. On the other hand, in the laboratory, determination of DIMP should be conducted using chromatographic techniques, especially when complex sample matrixes are involved. Moreover, given the unsurpassed confirmatory capacity of mass spectrometry to identify chemicals using their unique characteristic mass spectra, it is highly recommended to use the instrumental tandem GC-MS.

## Figures and Tables

**Table 1:** Formula and chemical and physical properties of di-isopropyl methyl phosphonate (DIMP) (information adapted from ref. [2]).

Substance Name and Formula	Properties
DIMP Di-isopropyl methyl phosphonate Methylphosphonic acid isopropyl ester C_7_H_17_O_3_P CAS#: 1445-75-6 EC#: 215-896-0	Molecular mass: 180.18 g mol^−1^ Boiling point: 121 °C @ 10 mm Hg Melting point: −25 °C Density of liquid DIMP: 0.976 g cm^−3^ @ 20 °C Refractive index: 1.4100 @ 30 °C Relative density of vapors: 6.25 (air = 1) Vapor pressure: 0.28 mm Hg @ 25 °C Volatility: 2800 mg m^−3^ @ 25 °C Proton affinity, PA: 931 kJ mol^−1^ Ionization energy: <10.60 eV Vaporization enthalpy: 57.6 kJ mol^−1^ Octanol–water coefficient (log K_ow_): 1.03 Solubility in water: soluble/miscible; 1500 mg L^−1^ Soluble in: methanol Acute toxicity: medium to low, with an LD_50_ between 500 and 1500 mg kg^−1^ (dependent on species) Oral RfD (reference dose), chronic: 0.08 mg kg^−1^ day^−1^ Conversion: 1 ppm_v_ = 7.50 mg m^−3^ (20 °C)

**Table 2:** Comparison between DIMP and the nerve agents sarin (GB) and soman (GD) [2].

	Sarin (GB)	Soman (GD)	DIMP
Formula	C_4_H_10_O_2_PF	C_7_H_16_O_2_PF	C_7_H_17_O_3_P
Molecular weight (M)	140.09	182.19	180.18
Vapor density (air = 1)	4.86	6.32	6.25
Vapor pressure [mm Hg] @ 25 °C	2.90	0.40	0.28
Volatility [mg m^−3^] @25 °C	22,000	4000	2800
1 ppm_v_ [mg m^−3^]	5.84	7.59	7.50
Acute toxicity: LD_50_ [mg kg^−1^]	24 (skin)	15 (skin)	500…1500 (oral)

**Table 3:** Concentrations of DIMP vapors in the standard atmospheres, correlated with the permeation rates (R) and the diluting air flowrate Q_air_:C_DIMP_ = R/Q_air_.

Permeation Rate (R) [ng min^−1^]	Diluting Air Flowrate (Q_air_) [cm^3^ min^−1^]	C_DIMP_ [mg m^−3^]	C_DIMP_ [ppb_v_]
45	3000	0.015	2
45	1200	0.038	5
90	1200	0.075	10
250	1100	0.23	30
900	1200	0.75	100
900	600	1.50	200
1900	500	3.75	500

**Table 4:** Summary of the quantitative results obtained with DIMP vapors using the LCD-3.2E hand-held ToF IMS instrument in the positive ion mode (three replicates were used for the peak height in order to calculate the standard deviation). Note: the positive reactant ion peak (RIP) appears at t_d_ = 4.66 ms and has a height of h_max_ = 8000 a.u.

C_DIMP_ [ppb_v_]	Drift Time Monomer, t_d_ [ms]	Peak Height Monomer, h_max_ [a.u.]	Drift Time Dimer, t_d_ [ms]	Peak Height Dimer, h_max_ [a.u.]
2	7.64	285 ± 30	10.32	19 ± 5
5	7.68	670 ± 57	10.32	17 ± 5
10	7.66	1240 ± 65	10.36	55 ± 8
30	7.60	1815 ± 74	10.28	145 ± 10
100	7.60	2430 ± 92	10.28	440 ± 23
200	7.60	2510 ± 104	10.28	520 ± 30
500	7.60	2280 ± 128	10.28	760 ± 42

**Table 5:** Reduced ionic mobilities (K_0_) calculated for the ions produced by DIMP.

Operation Mode	Ion Drift Time, t_d_ [ms]		Reduced Ion Mobility ^1^, K_0_ [cm^2^ V^−1^ s^−1^]	Reduced Ion Mobility ^2^, K_0_ [cm^2^ V^−1^ s^−1^]
	Pos RIP:	4.66	2.312	2.292
Positive	PIP 1 (monomer):	7.62	1.413	1.402
	PIP 2 (dimer):	10.28	1.044	1.039

^1^—Calculated by the IMS software TrimScan. ^2^—Calculated using the IMS cell constant (A): K_0_ = (A/t_d_).

**Table 6:** Resolving power of the LCD-3.2E IMS instrument for DIMP.

Ion Drift Time, t_d_ [ms]		Peak Width at Half Maximum, Δt_d_ [ms]	Resolving Power, R_IMS_
Pos RIP:	4.66	0.32	14.5
PIP 1 (monomer):	7.62	0.35	21.8
PIP 2 (dimer):	10.28	0.39	26.4

**Table 7:** Analytical figures of merit related to IMS detection of DIMP in the positive ion mode.

LoD [ppb_v_]	LoQ [ppb_v_]	Linear Range [ppb_v_]	Equation	R^2^	S [a.u./ppb_v_]
0.24	0.80	0.80–10	Y = 128.77·X + 15.242	0.9994	129.5

**Table 8:** Analytical figures of merit related to GC-MS detection of DIMP.

LoD [μg mL^−1^]	LoQ [μg mL^−1^]	Equation of Calibration Curve	R^2^	SD	Precision—RSD
0.21	0.62	Y = 1 × 10^8^X − 3 × 10^7^	0.9968	6.218·10^6^	1.21 (intra-day) 2.14 (inter-day)

**Table 9:** Quantification of DIMP using various IMS systems. RS—instrument equipped with a radioactive ionization source; NRS—instrument equipped with a non-radioactive ionization source.

Instrument	K_0_ [cm^2^ V^−1^ s^−1^]	Quant.	Ref.
DT-IMS and FAIMS [new FAT IMS] (RS)	1.47 (monomer) 1.07 (dimer)	4.80 ppb_v_ (monomer)	[20]
ToF IMS, model Airsense GDA-2, water chemistry (RS); DIMP concentration: 100 ppb_v_	1.54 (monomer) 1.08 (dimer)	-	[21]
ToF IMS with X-ray ionization and miniaturized IMS cell (l_d_ 40 mm) (NRS)	1.527 (monomer) 1.092 (dimer)	-	[22]
ToF IMS (RS) model CAM, with water vapor chemistry	1.55 (monomer) 1.13 (dimer)	-	[23]
ToF IMS (RS) model TeknoScan TSI-3000	1.468 (monomer) 1.042 (dimer)	-	[24]
ToF IMS (NRS) with 10.6 eV UV ionization	Only 1 peak 1.05	-	[25]
ToF IT IMS model Itemiser with heated cell (RS) and TD	1.52 (monomer) 1.03 (dimer)	15–35 pg	[26]
Three different ToF IMS devices (RS and NRS); water ionization chemistry	1.48–1.53 (monomer) 1.08–1.09 (dimer)	-	[27]
ToF IMS, model LCD-3.2E (NRS) Calibration: from 2 to 500 ppb_v_ Linear range: from 0.8 to 10 ppb_v_ Saturation: >500 ppb_v_	1.41 (monomer) 1.04 (dimer)	LoD 0.24 ppb_v_ LoQ 0.80 ppb_v_	this work

## Data Availability

The original contributions presented in the study are included in the article; further inquiries can be directed to the corresponding authors.
